# A word of caution: Spontaneous rupture of the noncoronary sinus of the Freestyle xenograft: Two cases and review of the literature

**DOI:** 10.1016/j.xjon.2022.06.012

**Published:** 2022-06-18

**Authors:** Ziyad Gunga, Salah Dine Qanadli, Guillaume Fahrni, Mario Verdugo-Marchese, Simon Koestner, Valentina Rancati, Zied Ltaief, Matthias Kirsch

**Affiliations:** a Department of Cardiac Surgery, Centre Hospitalier Universitaire Vaudois, Lausanne University, Lausanne, Switzerland; b Department of Radiology, Centre Hospitalier Universitaire Vaudois, Lausanne University, Lausanne, Switzerland; c Department of Anesthesiology, Centre Hospitalier Universitaire Vaudois, Lausanne University, Lausanne, Switzerland

**Figure:**
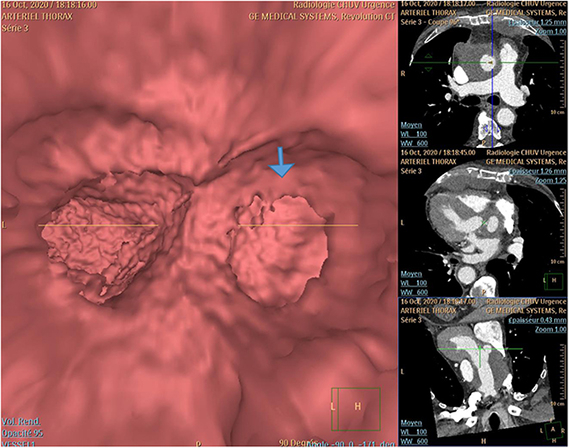
Endovascular virtual reality angiography highlighting the fenestration (*arrow*) of the noncoronary sinus seen from the inside (case 2).

**Central Message**

Freestyle bioprosthesis is an ideal substitute for aortic root replacement. Rupture of the Freestyle has been rarely described. We present 2 cases of spontaneous rupture of the noncoronary sinus.

The porcine stentless Freestyle xenograft (Medtronic Inc) has proved to be an ideal substitute for aortic root replacement (ARR) as it provides excellent hemodynamics and durability.^1^^,^^2^ Nonetheless, like other bioprostheses, structural valve deterioration is observed in the long term, mostly due to aortic cusp tears. However, spontaneous rupture of the Freestyle xenograft aortic wall has also been reported.^3^^,^^4^ We present 2 patients who developed pseudoaneurysm after ARR using a Freestyle xenograft, related to spontaneous rupture of the noncoronary sinus (NCS) of Valsalva.

## Case 1

In November 2018, an 80-year-old male patient with a history of aortic root dilation had a complete ARR using a 25-mm Freestyle xenograft. In August 2020, the patient was hospitalized for acute lung edema related to massive aortic valve insufficiency due to deformation of the xenograft by a pseudoaneurysm. Intraoperative findings noted a tear of the upper portion of the NCS of the Freestyle xenograft. This part was trimmed, and a reconstruction of the sinotubular junction was achieved using a 24-mm Dacron graft, strengthened by an extraluminal pericardial patch.

In October 2020, a computed tomography scan highlighted a recurring pseudoaneurysm, triggering a third operation. A 1-cm rupture of the NCS was identified, without sign of infection. The Freestyle xenograft was trimmed, leaving the annulus, and a redo ARR was performed using a 23-mm Freestyle xenograft. The patient died 1 week later due to sudden cardiac arrest secondary to acute hypoxemia following bronchoaspiration. Transthoracic echocardiogram confirmed the absence of pericardial tamponade.

## Case 2

A 72-year-old female patient with a history of aortic valve replacement in 2018 was admitted emergently for type A dissection in February 2020. ARR using a 25-mm Freestyle xenograft was performed, associated with hemiarch replacement.

In November 2020, the patient was reoperated for severe tricuspid insufficiency, and operative findings pinpointed a small pseudoaneurysm on the suture line between the distal portion of the Freestyle xenograft and the Dacron prosthesis. This 2-mm orifice was sutured and strengthened with heterologous pericardium and BioGlue (CryoLife, Inc).

**Figure 1:**
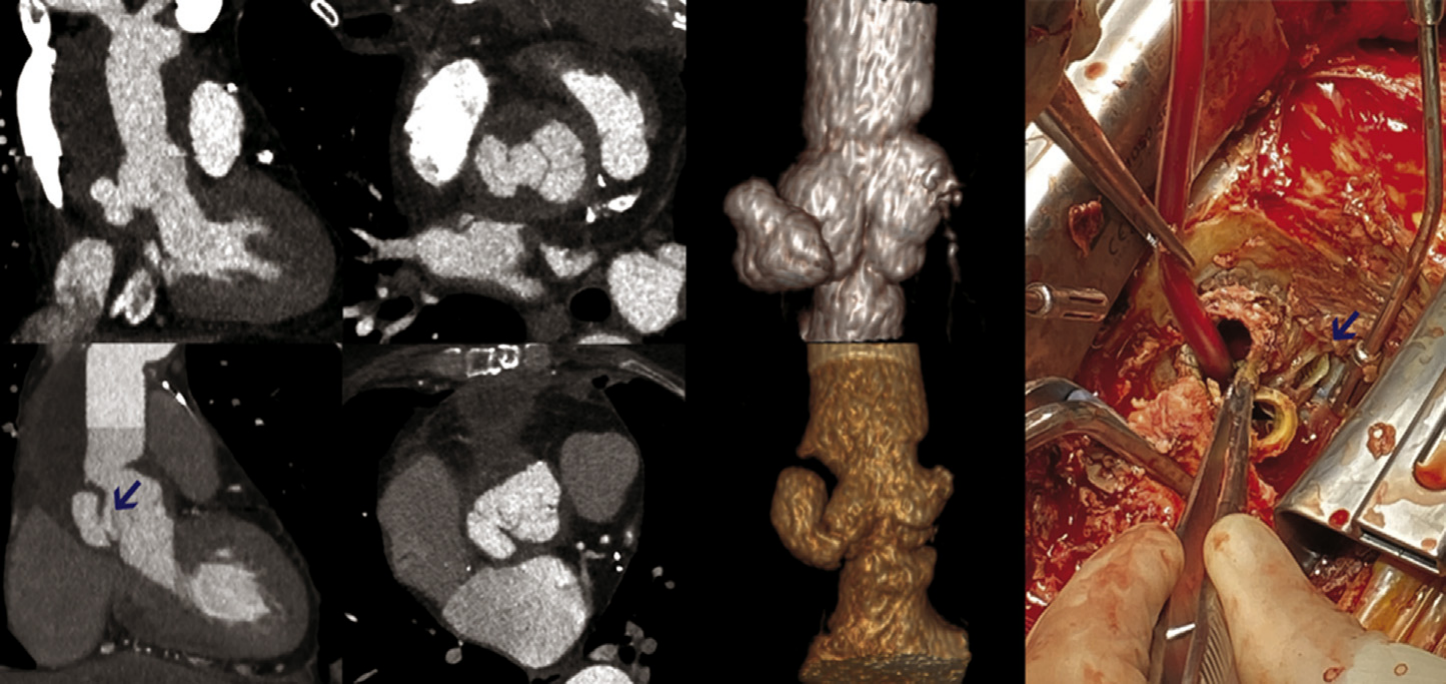
*Left*, Computed tomography and 3-dimensional reconstruction (cases 1 and 2); *right*, operative image rupture of the noncoronary sinus (case 2, *arrow*).

In October 2021, an acute thoracic pain triggered a computed tomography scan that showed a large circulating pseudoaneurysm of the aortic root originating from a tear in the NCS of the Freestyle xenograft (Figure 1). Intraoperative findings confirmed the rupture in the central part of the NCS, without sign of infection (Video 1). A redo ARR using a 25-mm Freestyle was performed. The patient had an uneventful recovery. Both patients gave written informed consent for the use of their data for publication use.

## Comment

The Freestyle xenograft is the only currently commercially available aortic root xenograft. It provides excellent hemodynamic results in terms of postoperative transvalvular gradients, effective orifice area, and regression of left ventricular hypertrophy. Furthermore, excellent durability has been reported by several studies, with 1-year freedom from reoperation rates of more than 90%.^1^^,^^2^

However, early- and mid-term structural valve deteriorations have been outlined. In addition to cusp tears, the aortic wall of the Freestyle xenograft seems also vulnerable, as highlighted by the present cases. Six similar cases of spontaneous rupture of the Freestyle xenograft wall have been previously reported, and are summarized in Table 1. Interestingly, the rupture occurred most frequently in the noncoronary sinus of the xenograft.

**Table 1:** A review of literature in PubMed highlighting 6 cases of noncoronary sinus perforation of the Freestyle Medtronic bioprosthesis

Study	Age, y	Sex	Surgery	Etiology	Time after surgery, mo	CT scan diagnosis	Operative findings	Fenestration site	Reoperation	Infection
Kameda et al^5^	71	Female	Full root Freestyle + AAR (Dacron)	Aneurysm + AR	15	Aortopulmonary fistula (aortic wall rupture)	2 holes: 3 × 7 mm/3 × 5 mm	Noncoronary sinus	Strengthened with patch and AAR with Dacron graft	No evidence
Ozaki et al^3^	63	Male	Full root Freestyle 23 mm	Aneurysm + AR	51	Pseudoaneurysm (aortic wall rupture)	1 hole: 15 × 5 mm	Noncoronary sinus	Full root Freestyle	No evidence
Ozaki et al^3^	62	Male	Full root Freestyle 21 mm	Aneurysm + AR	20	Pseudoaneurysm (aortic wall rupture)	1 hole: 15 mm	Noncoronary sinus	Full root Freestyle 21 mm	No evidence
Ozaki et al^3^	63	Female	Full root Freestyle 25mm + arch	Aneurysm + AR	17	Pseudoaneurysm (aortic wall rupture)	2 holes: 20 mm	Non-coronary + left sinuses	Full root Freestyle 25 mm	No evidence
Ozaki et al^3^	63	Female	Full root Freestyle 25 mm + arch	Bentall redo	20	Pseudoaneurysm (aortic wall rupture)	3 holes: 20 mm	Noncoronary + left sinuses	AVR with CE 23-mm with AAR 28-mm straight graft	No evidence
Kitamura et al^6^	65	Male	Full root Freestyle 25 mm	Aneurysm + AR	18	Pseudoaneurysm (aortic wall rupture)	1 hole	Noncoronary sinus	Composite graft	No evidence

*CT*, Computed tomography; *AAR*, ascending aortic replacement; *AR*, aortic regurgitation; *AVR*, aortic valve replacement; *CE*, Carpentier-Edwards.

Since neither of the cases has been related to an infectious origin, we retain 3 hypotheses to explain these clinical observations. Ozaki and colleagues^3^ suggested a weakness in the transitional zone between annular collagen fibers and the aortic elastic wall. Similarly, using biomechanical analyses, Takaya and colleagues^4^ noted that NCS was significantly thinner and less elastic than the other sinuses, hence inherently more vulnerable and at greater risk of tissue failure.

Alternatively, traumatic manipulation of the Freestyle xenograft, such as pinching of the prosthetic wall, could contribute to its subsequent failure. In our practice, we used to invaginate the Freestyle into the left ventricular outflow tract to facilitate tying of the proximal suture line. In view of the present cases, this practice cannot be recommended anymore.

Finally, a toxic factor could also be incriminated. Indeed, Kazui and colleagues^7^ have reported that BioGlue surgical adhesive might occasionally cause severe injury to aortic tissue, such as excessively thinning of aortic tissue at the site of adhesive application, hence inciting pseudoaneurysm formation.

We underline the importance of additional multidimensional imaging in the follow-up to depict structural aortic abnormalities after Freestyle implantation, insufficiently unveiled by TEE as suggested by Dagnegård and colleagues.^8^

## Conclusions

Spontaneous rupture of the NCS constitutes a rare hazard after ARR using the Freestyle xenograft. Atraumatic handling of the bioprosthesis and avoidance of potentially toxic surgical adhesives should be recommended. Meticulous follow-up should be devised to detect this potential complication.

## Supplementary Data

Video 1 Perioperative findings in case 2. We have highlighted the hole in the noncoronary sinus (NCS) of the Freestyle bioprosthesis, a circular defect of 1 cm. A solidified BioGlue remnant was removed from the pseudoaneurysm adjacent to the NCS of the Freestyle. Video available at: https://www.jtcvs.org/article/S2666-2736(22)00283-2/fulltext.
