# Supplementary figures and images for: A word of caution: Spontaneous rupture of the noncoronary sinus of the Freestyle xenograft: Two cases and review of the literature
Source: JTCVS Open. 2022 Jun 18;11:59–61. doi: 10.1016/j.xjon.2022.06.012 (PMC9510863; doi:10.1016/j.xjon.2022.06.012)

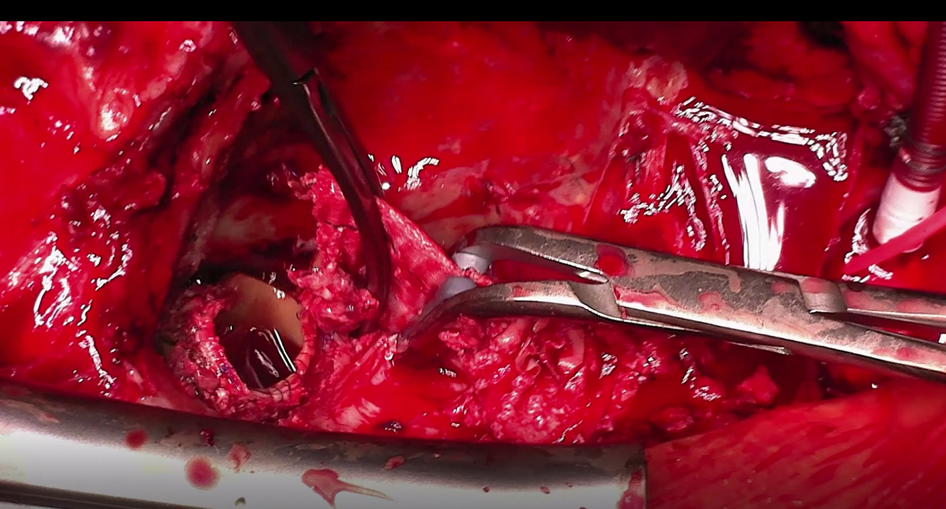

Supplement: Video 1 — Perioperative findings in case 2. We have highlighted the hole in the noncoronary sinus (NCS) of the Freestyle bioprosthesis, a circular defect of 1 cm. A solidified BioGlue remnant was removed from the pseudoaneurysm adjacent to the NCS of the Freestyle. Video available at: https://www.jtcvs.org/article/S2666-2736(22)00283-2/fulltext. [file fx2.jpg]
